# The Role of Spermidine and Its Key Metabolites in Important, Pathogenic Human Viruses and in Parasitic Infections Caused by *Plasmodium falciparum* and *Trypanosoma brucei*

**DOI:** 10.3390/biom13050803

**Published:** 2023-05-09

**Authors:** Annette Kaiser

**Affiliations:** Medical Research Centre, University of Duisburg-Essen, Hufelandstrasse 55, 45147 Essen, Germany; kaiser@microbiology-bonn.de

**Keywords:** spermidine, viral and parasitic infections, drug development

## Abstract

The triamine spermidine is a key metabolite of the polyamine pathway. It plays a crucial role in many infectious diseases caused by viral or parasitic infections. Spermidine and its metabolizing enzymes, i.e., spermidine/spermine-N^1^-acetyltransferase, spermine oxidase, acetyl polyamine oxidase, and deoxyhypusine synthase, fulfill common functions during infection in parasitic protozoa and viruses which are obligate, intracellular parasites. The competition for this important polyamine between the infected host cell and the pathogen determines the severity of infection in disabling human parasites and pathogenic viruses. Here, we review the impact of spermidine and its metabolites in disease development of the most important, pathogenic human viruses such as SARS-CoV-2, HIV, Ebola, and in the human parasites *Plasmodium* and *Trypanosomes*. Moreover, state-of-the-art translational approaches to manipulate spermidine metabolism in the host and the pathogen are discussed to accelerate drug development against these threatful, infectious human diseases.

## 1. Introduction

### The Biosynthesis and Metabolism of the Triamine Spermidine in the Human Host Cell and Its Regulation

**Figure 1:**
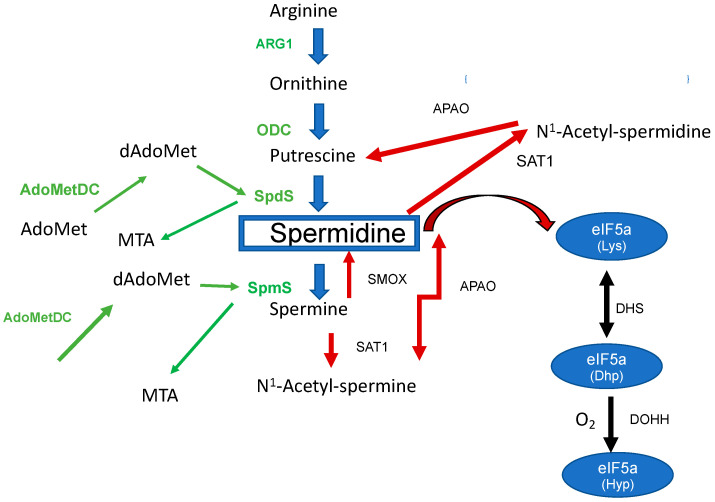
The biosynthesis of spermidine and its metabolism in the mammalian host. Enzymes involved in the biosynthesis are labeled in green: Arg1 (arginase), ODC, SpdS, SpmS, AdoMetDC (S-Adenosylmethionine decarboxylase), dAdoMet (decarboxylated S-Adenosylmethionine), and Methylthioadenosine (MTA). Spermidine metabolizing enzymes are marked with red arrows: PAOX, SAT1, and Spermine oxidase (SMOX). The right part of the figure shows the biosynthesis to hypusine: DHS (deoxyhypusine synthase) and DOHH (deoxyhypusine hydroxylase). Legends within Figure 1 were left out according to the reviewer’s (1) recommendation.

The triamine spermidine is a biogenic basic polyamine that is essential for the human host cell. In eukaryotes, polyamines can be formed either from the amino acids ornithine (Orn) or arginine (Arg). However, the biosynthesis of PAs exclusively starts from Arg in mammalian cells (Figure 1) with arginase (Arg1) catalyzing the reaction from Arg to Orn.

The second step of the polyamine pathway [1] (Figure 1) is initiated by the decarboxylation of Orn to Put by ornithine decarboxylase (ODC). Within the next step, spermidine synthase (SpdS) transfers an aminopropyl moiety from decarboxylated S-adenosylmethionine (dSAM) to Put which results in the formation of spermidine (Spd). Spermine (Spm) is formed from Spd under the catalysis of spermine synthase (SpmS). In this reaction an aminopropyl moiety is transferred from dSAM to form Spm. Collectively, there are two aminopropyltransferases which catalyze the addition of aminopropyl groups sequentially.

Apart from its function as a main and central PA in the mammalian host, Spd can be catabolized to metabolites which play an important regulatory role in the defense against pathogens (Figure 1). Spd is degraded to N^1^-Acetylspermidine by spermidine-N^1^-acetyltransferase (SAT1) and can finally be oxidized to Put by polyamine oxidase (PAOX) [1].

Likewise, Spm has different metabolic routes. It can be interconverted to Spd by spermidine oxidase (SMOX), or it can be converted to N^1^-Acetylspermine by (SAT1) and finally be oxidized to spermidine by polyamine oxidase (PAOX) [1].

Polyamine pools and intracellular levels of polyamines in mammals are regulated by a network of biosynthesis, export, degradation, and transport [2] mechanisms. One central point of regulation is ODC in the biosynthetic pathway of PAs. ODC is regulated at different levels, i.e., at the transcriptional level by Myc [3] and post-translationally by antizyme [4] which prevents ODC dimerization and targets the protein for proteosomal de-gradation.

A second, critical, point of PA pool regulation is the catabolism of the biological significant triamine Spd. Catabolism of Spd is performed by SAT1, the rate limiting step which mediates the transfer of acetyl CoA to result in acetylspermidine followed by PAOX. SAT1 is regulated at different levels. At the level of transcription, two transcription factors, i.e., erythroid 2-related factor 2 (Nrf-2) and its co-factor polyamine modulated factor (PMF-1) bind to the polyamine-response element in the promotor region of the *SAT1* gene [5]. Moreover, under certain environmental stimuli, a network of different transcription factors, such as TNF-α can be functional to induce binding to the specificity protein element (SP-element) within the promotor region of the *SAT1* gene to keep a basal level of transcription [6]. At the post-translational level, SAT1 activity is regulated by dimerization of the two functional dimers of the protein [7].

SMOX is a highly inducible protein responsible for interconversion of Spm to Spd. Upon tissue injury and inflammation, SMOX activity is upregulated [8]. Progression of inflammation is further supported by reactive metabolites such as H_2_O_2_, 3-aminopropanal, and acrolein which are formed by the SMOX catalyzed reaction [9]. SMOX can also be induced by pro-inflammatory cytokines such as interleukin-6 or TNF-α [10] due to the occurrence of polyamine responsive elements and multiple responsive elements in the SMOX promotor region [11].

Spd is an important substrate in a two-step reaction leading to the post-translational modification of hypusine at lysine 50 in eukaryotic initiation factor 5A (eIF5A) [12]. This modification is unique for eIF5A. In the first reaction, an aminobutyl moiety is transferred from spermidine to lysine 50 in eIF5A to generate a deoxyhypusine residue after NAD-dependent cleavage of spermidine and formation of an enzyme-butylimine intermediate [13]. In the second step, deoxyhypusine hydroxylase completes hypusine biosynthesis, introducing a hydroxyl group at carbon 9 of the deoxyhypusine residue side chain to form hypusine [14]. Apart from processing mRNAs with a proline motif [15], a variety of functions can be attributed to the modified protein. The role in the pathogenesis of different infectious diseases, its impact on diabetes, immune cells, and neurological disorders [16,17,18,19] has been recently recognized.

The recent outbreaks of severe respiratory syndrome corona virus 2 (SARS-CoV-2) and the resistance against infectious diseases, such as Malaria and Trypanosomiasis, have highlighted significant limitations in our ability to control and treat them. In this review, the potential of the triamine Spd and its metabolites is outlined in the process of infection and treatment of the most important disabling human viruses and parasites.

## 2. The Impact of Spermidine and Its Metabolites in Infection with Human Pathogenic Viruses

### 2.1. Dysregulation of Spermidine Metabolism Determines SARS-CoV-2 Infection

**Figure 2:**
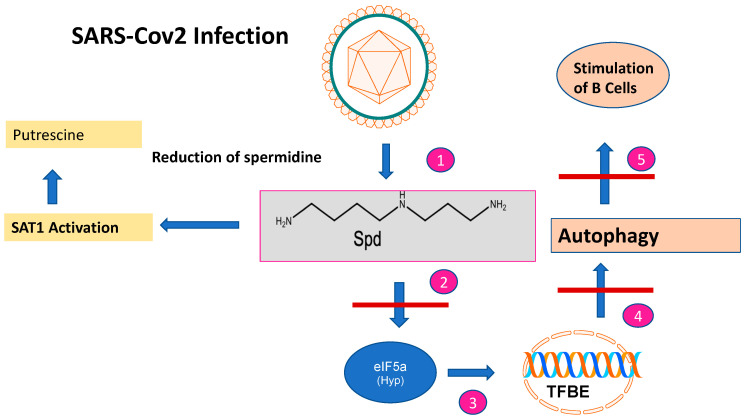
The impact of SARS-CoV-2 infection on spermidine metabolism. After SARS-CoV-2 infection, the spermidine concentration decreases (step 1). This is caused by SAT1 activation and a metabolic shift to putrescine. The decreased spermidine concentration affects EIF5A leading to reduced EIF-5A formation (step 2) and subsequent translation of the TFBE transcription factor (step 3). This results in reduced transcription of genes involved in autophagy (step 4) and stimulation of B cells involved in immune responses (step 5).

**Figure 3:**
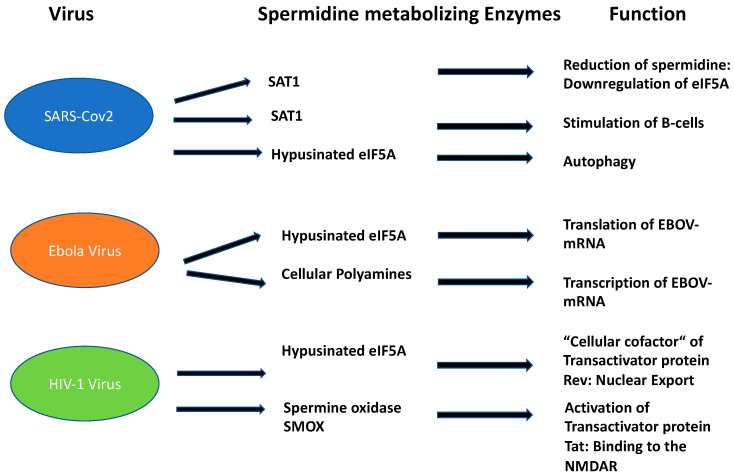
Function of spermidine and its metabolizing enzymes in the three most important disabling human viruses.

The role of Spd and its metabolites in mammalian viral propagation is diverse. Their main functions during infection can be attributed to viral entry, transcription, replication, and virion packaging. The impact of Spd in infections has been recently demonstrated in an in-depth metabolic study for SARS-CoV-2 [20] during the pandemic from 2020 to 2022. SARS-CoV-2 is an RNA virus that belongs to the coronavirus family. According to our current knowledge, it is zoonotically transmitted to humans, similar to the SARS virus. For replication in the human host, it needs binding of its spike proteins to the Angiotensin converting enzyme receptor 2 (ACE). The entrance of SARS-CoV-2 into the human host cell is only possible with the host-specific protease TMPRSS2. SARS-CoV-2 is mainly transmitted by droplet infection and can enter the human host via the respiratory tract, the eyes, the nose, or the mouth. Primarily, the respiratory tract is affected but other organs can also be affected. Infected people with mild symptoms, or even without, can transmit the disease [21]. Upon infection, SARS-CoV-2 causes a dysregulation of important key metabolites in the human host cell. The virus reduces autophagy which causes degradation of macromolecules into their monomers, such as amino acids and fatty acids, after fusion with acidic lysosomes. Spd plays a key role in these processes since it is an inducer of autophagy [22] (Figure 2): (i) The acetylated forms of Spd promote the export of polyamines and reduce viral propagation [23]; (ii) moreover, Spd as a building block in hypusine formation of eIF5A, activates translation of the transcription factor TFBE which induces a set of proteins involved in autophagy [24]; (iii) interestingly, *SAT1* is transcriptionally upregulated during SARS-CoV-2 infection in vitro and in vivo. This result was confirmed by metabolome analysis which revealed an increase in putrescine levels. This shift to putrescine reduces the activity of hypusinated eIF5A and the translation of TFBE m-RNA [20]. Since SAT1 stimulates B cells, manipulation of SAT1 dynamics might be a way to circumvent the immune response of the host by SARS-CoV-2 infection. Translational experiments based on single-nucleus and single-cell sequencing of patient- derived lung and mucosal samples, revealed differential transcriptional regulation of autophagy and immune genes depending on SARS-CoV-2 replication levels [20]. Conclusively, targeting autophagy pathways is an attractive treatment option for SARS-CoV-2 infection (Figure 3). Exogenous supplementation with the PAs Spd, Spm, and an autophagy inhibitor MK-2206, as well as the phagolysosome inhibitor niclosamide, inhibited SARS-2 propagation with in vitro IC_50_ values of 136.7, 7.67, 0.11, and 0.13 μM, respectively [20].

### 2.2. The Role of Hypusinated EIF-5A and PAs in Ebola Propagation

Ebola virus (EBOV) is a nonsegmented negative-sense RNA virus in the Filoviridae family. It causes Ebola virus disease (EBOVD). EBOVD starts from a single case of probable zoonotic transmission; while in the next step, human to human transmission by infected body fluids follows [25]. Ebola virus infections belong to the deadliest viruses, with fatality rates between 40% and 90%.

In 2019, the first vaccine against EBOVD was approved by the European Medicines Agency [26]. Apart from remdesivir [27], two monoclonal antibodies targeting the envelope glycoprotein showed promising results [28]. The Ebola genome has a small size of 19 kb, expressing seven structural proteins and seven non-structural proteins. Seven genes encode the following proteins: *NP* nucleoprotein, VP35 polymerase cofactor, VP40 matrix protein, GP glycoprotein and secreted glycoproteins, VP30 transcriptional activator, VP24 RNA complex-associated protein, and L large protein. These viral proteins are responsible for the transcription and replication of the EBOV genome before the assembly and egress of new viral particles. Polyamines from the host cell are also essential for the replication of EBOV [29]. Most viruses lack an intact polyamine pathway, barring one exception, i.e., PBC-V1 virus [30]. This virus encodes a complete polyamine pathway in its ds DNA genome and infects a chlorella-like green alga. Therefore, in the case of EBOV, the question arises whether cellular PAs from the human host are necessary at the level of translation such as spermidine in modified eIF5A or in multiple stages of gene expression due to their diverse cellular functions (Figure 3). A recent study [30] performed with a replicase-deficient minigenome from EBOV demonstrated the requirement of hypusinated eIF5A of the infected host cell. However, inhibition with N^1^-guanyl-1,7-diaminoheptane (GC7), an inhibitor of DHS, did not show an impact on the formation of EBOV transcripts, but rather on the protein level. In contrast, when reporter m-RNA constructs from hypusine deficient cells were transfected into host cells in the absence of GC7 inhibitor with normal function of eIF5A, the EBOV-transcripts were normally translated [31]. Conclusively, hypusination of the host cell is essential for translation of EBOV-transcripts. In contrast, depletion of polyamines with the ODC inhibitor DL-α-Difluoromethylornithine (DFMO) prevents the synthesis of polymerase induced EBOV-mRNA. This effect could be reversed by supplementation with exogenous polyamines [31]; suggesting that eIF5A and exogenous PAs from the human host fulfill two different functions in the replication of Ebola virus. Thus, repurposing of these inhibitors to interrupt polyamine interaction between the host cell and EBOV replication might be a novel therapeutic option.

### 2.3. Hypusinated eIF5A Is a Cofactor of HIV-1 Rev Regulatory Protein

More than 76 million people worldwide are infected with the human immunodeficiency virus (HIV) which causes acquired immune deficiency syndrome (AIDS). There are now 37 million people living with the infection [32] due to a highly effective antiretroviral therapy (HAART). Human immunodeficiency is caused by a collection of viruses, with human immunodeficiency virus 1 (HIV-1) being the most prevalent and HIV-2 being less pathogenic. HIV-1 is an RNA virus of the genus Lentivirus which belongs to the family of *Retroviridae*. The disease leads to progressive loss of CDC4^+^ cells, causing infectious and immune abnormalities and oncological complications. Meanwhile, antiretroviral drugs are highly efficient for patients who can access and adhere to them, thus leading to durable and probable long-life suppression of viral replication. HIV is a retrovirus that integrates its DNA into the human genome. The main receptor for HIV virus is the CD4 receptor together with two chemokine receptors, CCR5 and CXCR4. These receptors are expressed on T lymphocytes, macrophages, and monocytes. After fusion and uncoating, the RNA is reverse transcribed into DNA. Subsequently, HIV DNA is transcribed to viral mRNA, after import in the nucleus. These HIV mRNAs are then exported to the cytoplasm where translation occurs to make viral proteins and eventually mature virions. Each step of the HIV life cycle, i.e., HIV entry, reverse transcription, integration, and protein maturation, can be targeted by antiretroviral drugs. EIF5A plays an important role in HIV-1 replication since it is a “cellular cofactor” of the HIV-1 trans-activator protein Rev (Figure 3) [33]. Rev is essential for HIV-1 protein expression. Within the *Rev* gene, a nuclear localization signal is encoded which is responsible for its localization in the nucleus. The function of Rev is the nuclear export of spliced and incompletely spliced mRNAs [34]. The absence of Rev leads to disturbances in nuclear export, i.e., mRNAs remain in the nucleus without translation.

Since hypusinated EIF-5A is a “cellular cofactor” of the trans-activator protein Rev, clinical investigations have been performed to target deoxyhypusine synthase (DHS), the first enzyme of the hypusine pathway, for antiretroviral therapy [35]. Inhibition of human DHS with the small molecule CNI-1493, a polyguanylghydrazone, efficiently suppressed HIV replication in macrophage- and T cell-tropic laboratory strains, clinical isolates, and viral strains with high-level resistance to inhibitors of viral protease and reverse transcriptase. Moreover, comparable antiretroviral effects were achieved with RNA interference, suggesting that DHS is a promising target for antiretroviral therapy. Alternatively, the depletion of spermidine biosynthesis by inhibition of S-adenosylmethionine decarboxylase (AdoMetDC) [36], a branch point enzyme that produces dAdoMet, led to suppression of modified eIF5A and Rev inhibition, respectively. Collectively, the data show that spermidine depletion is an alternative strategy to affect hypusinated eIF5A and its “cellular cofactor” Rev to interrupt HIV-1 replication. Apart from hypusinated eIF5A, spermine oxidase (SMOX) [37] is also involved during HIV infection. In HIV-1 infected brain tissues, oxidative stress can be induced by HIV-1 secreted proteins such as HIV-1 Tat, leading to neuronal death through interaction with the subtype of the polyamine sensitive *N*-methyl-d-aspartate receptor (NMDAR). This phenomenon is known as HIV-associated dementia (HAD) and appears together with motorneurological disorders, which appear in a subset of patients. Tat-induced oxidative stress in a neuroblastoma cell line led to the elevation of SMOX activity and reduction in spermine levels. This effect could either be reversed by supplementation with the antagonist MK-801, an inhibitor of the NMDAR site, or with N-acetylcysteine, a scavenger of H₂O₂. Conclusively, these data show an important role for polyamine catabolism-induced H₂O₂ formation which elicits the binding of Tat to the polyamine sensitive NMDAR [37].

## 3. Spermidine as a Hallmark in the Apicomplexan Parasite *P. falciparum*

Apicomplexan parasites represent a phylum with an apical complex; a conical structured organelle at the apical end of the cell that enables the parasite to penetrate the host by micronemes, rhoptries, conoid, and polar rings [38]. The apicomplexan parasites of medical importance include *Plasmodium* spp. (causing malaria), *Cryptosporidium parvum* (causing Cryptosporidiosis), *Babesia* sp. (causing Babesiosis), and *Toxoplasma gondii* (causing Toxoplasmosis). Most of the apicomplexan parasites are sporozoans but some of them also produce oocytes. In this review we focus on *P. falciparum* since it is the deadliest human parasite.

**Figure 4:**
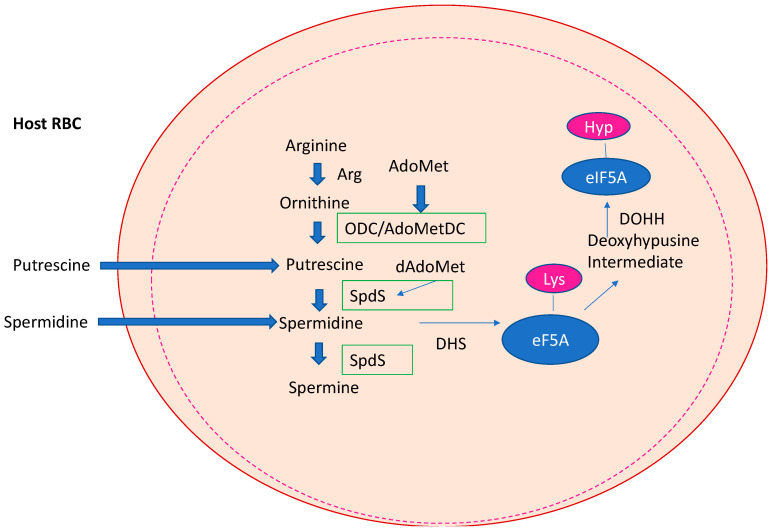
*De novo* biosynthesis of polyamines (PAs) in *Plasmodium falciparum*. *Plasmodium* has a core PA pathway with ornithine decarboxylase (ODC), S-adenosylmethionine decarboxylase (AdoMetDC), and spermidine synthase (SpdS). A bifunctional AdoMetDC and a SpS producing Spm are peculiar for this pathway in *Plasmodium*. The parasite is also able to use Put and Spd from the salvage pathway of the infected host red blood cell (RBC). Hypusine biosynthesis is highly conserved. Deoxhypusine synthase (DHS) catalyzes the transfer of the aminopropyl moiety to lysine 50 in the precursor protein and deoxyhypusine hydroxylase (DOHH) introduces the hydroxyl group to carbon 9 in the side chain.

Almost 50 years after the establishment of the WHO Global Malaria Programme, malaria remains a major global health problem with an estimated 627,000 deaths in 2020, according to a WHO report published in 2021 [39]. Although the number of deaths decreased between 2000 and 2019, SARS-CoV-2 refueled the rate of infection. Moreover, the death rate of children under the age of five remained at 72%. The development of the RTS,S vaccine against the parasite’s circumsporozoite protein was a milestone although it showed only a short-lived immune protection in phase III clinical trials in different African countries [40]. Therefore, next-generation vaccines with prolonged immune protection are necessary and are beyond the horizon [41]. Eradication of malaria is a major problem, since its causative agent, *P. falciparum*, has a complex life cycle and can remain latent within the human host [42]. Conclusively, eradication of malaria will only be successful with a combined therapy of vaccination and small molecules due to the developing resistance against registered drugs. *Plasmodium* ssp. perform de novo polyamine biosynthesis but also use a salvage pathway for the uptake of Put and Spd [43,44] in the infected erythrocyte. The first reports about highly specific Put and Spd carriers demonstrated novel transporters with different kinetic parameters compared to transporters in the uninfected erythrocyte [43]. Later, radioisotope flux techniques proved the uptake of Put and Spd in a temperature dependent process from the infected erythrocyte [44]. *Plasmodium* is the only apicomplexan parasite which contains a full set of key enzymes involved in the biosynthesis of spermidine (the core pathway), i.e., ODC, AdoMetDC, and SpdS (Figure 4), and a conserved pathway leading to the posttranslational modification of hypusine in eIF5A. Catabolic enzymes such as SAT1, APAO, and SMOX are absent in the parasite. Peculiarities of the core biosynthetic route leading to the triamine Spd are a bifunctional ODC/AdoMetDC [45] and the occurrence of a SpdS which can catalyze the formation of Spm [46]. Spd is an essential substrate for hypusine formation in *Plasmodium* [47]. Both enzymes of the hypusine pathway have been cloned and characterized and have shown to be vital genes in murine blood-stages [48] using reverse genetics similar to homologous recombination techniques, suggesting the importance of the pathway for infection. Moreover, a recent targeting of SpdS in *P. yoelii,* by deletion/complementation analyses, demonstrated the gene to be essential for blood-stage growth [49]. Interestingly, SpdS was located in the mitochondria of blood-stages and sporozoites pinpointing its role as a drug candidate of interest.

Although a variety of specific small molecule inhibitors have been identified to block spermidine [50] and hypusine biosynthesis [51,52] in *Plasmodium*, a continuous strategy is still missing to develop parasite-specific efficient inhibitors to the clinical phase. A major problem with the less efficient SpdS inhibitors resulted from a sequential order of substrate binding to the protein. First, the aminopropyl binding site must be occupied with dAdoMet; next, putrescine must be bound to the putrescine binding site; finally, a specific ligand can dock to the cavity of SpdS [50]. In the case of the hypusine pathway, the identified DHS inhibitors were spermidine mimetics similar to N^1^-guanyl-1,7-diaminoheptane GC7 with undesired side effects [53] due to a lack of target selectivity. Attempts to inhibit DOHH were based on an iron complexing strategy with known iron chelators like ciclopirox or zileuton which were used as repurposing drugs [52]. However, current crystallization experiments will hopefully foster the development of specific inhibitors against DOHH from the parasite.

## 4. Spermidine Biosynthesis and Its Metabolizing Pathways in the Trypanosomatids Are Unique

**Figure 5:**
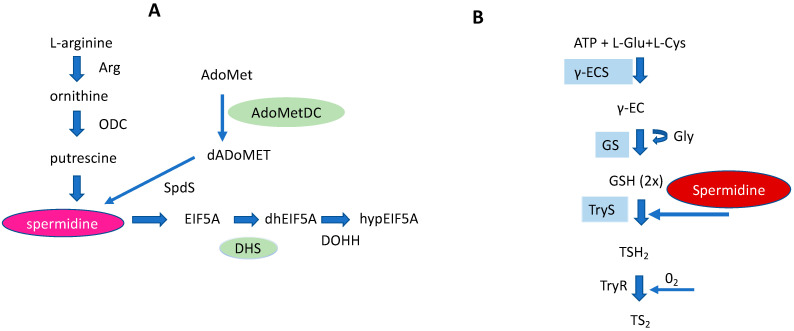
Schematic representation of a core PA pathway (**A**), and a Trypanothione pathway (**B**) in Trypanosomatids. In both pathways spermidine represents an important key metabolite. Pseudoenzymes, i.e., DHS and AdoMetDC of a core PA pathway are marked in green. The right part of the figure: Cys (cysteine) and Glu (glutamate) are covalently linked by γECS (gamma-glutamylcysteine synthetase) to form γEC (gamma-glutamylcysteine). Gly (Glycine) is bound to γEC by glutathione synthetase (GS) producing glutathione (GSH). Finally, trypanothione synthetase (TryS) synthesizes T(SH)_2_ by binding two GSH molecules to a Spd molecule. Trypanothione reductase (TyrR) catalyzes oxidized T(SH)_2_ reduction to TS_2_.

The trypanosomatids cause a variety of insect-born pathogenic, infectious, human diseases such as human African trypanosomiasis (*T. brucei*), Chagas disease (*T. cruzi)*, and Leishmaniasis (*Leishmania* species). According to WHO reports [54], an estimated 18 million people are infected worldwide. In trypanosomatids, a core PA pathway and a trypanothione pathway exist as unique metabolic pathways [55]. They both utilize spermidine as a key metabolite (Figure 5). Most of the enzymes of both pathways are essential for survival, growth, and infectivity. One peculiar feature of the core PA pathway is the occurrence of pseudoenzymes that evolved from enzymes into regulators [56]. In this context, AdoMetDC and DHS are outstanding examples (Figure 5). However, trypanosomatids lack catabolizing enzymes such as SMOX, SSAT, and APAO although some catabolites produced by these enzymes appear in the parasite. This review has a main focus in *T. brucei*.

The Trypanosome species differ in their requirement for polyamines. This might be explained by their life habitat. *T. brucei* causes African sleeping thickness and is an extracellular parasite which replicates in the bloodstream and strongly depends on exogenous polyamines apart from the natural PA pathway. In *T. brucei,* ODC and AdoMetDC (Figure 5) maintain the level of spermidine which is also necessary for trypanothione biosynthesis as a redox cofactor. Two AdoMetDC paralogs [57] exist in the parasite, forming a heterodimer which regulates the spermidine biosynthetic pathway. The inactive AdoMetDC paralog (termed prozyme) plays a role as an allosteric activator of the functional heterodimer. Silencing constructs and conditional knockouts in the bloodstream demonstrated that prozyme is essential and indispensable for parasite survival and infection [57]. Apart from ODC and AdoMetDC, SpdS is essential in *T. brucei* bloodstream stages [58]. The protein has been validated as a druggable target by RNAi constructs. Interestingly, exogenously supplied spermidine does not rescue growth in strains with a silenced SpdS, suggesting the absence of a spermidine specific transporter [59]. Surprisingly, N-acetylspermidine has not been detected in *T. brucei* as a metabolite of spermidine, while N-acetylputrescine and N-acetylornithine have been identified by isotope labelling of glucose [60]. However, the function and formation of these catabolites remain obscure. Spermine is absent in *T. brucei*. The prozyme paradigm can also be attributed to DHS from *T. brucei*. Within the *T. brucei* genome, two DHS paralogs are encoded [61]. One paralog has an impaired catalytic activity while the other paralog does not show any enzymatic activity. Coexpression of both proteins resulted in a heterotetramer with 3000-fold activity. Conditional knockouts showed that both DHS proteins are essential for growth in vitro and in murine models.

*T. cruzi* and *Leishmania* are intracellular parasites which can utilize spermidine from the human host cell. Indeed, this is true for *T. cruzi* which has lost its ODC [62]. Although intact AdoMetDC [63] and SpdS [64] occur, *T. cruzi* strictly depends on putrescine and spermidine from the infected host cell by transport mechanisms. Catabolic enzymes for spermidine and spermine such as PAO or SSAT are missing [65]. Hitherto, there are no reports about the molecular cloning of *dhs* and *dohh* genes in *T. cruzi.*

## 5. The Impact to Develop Inhibitors against Enzymes Involved in Spermidine Biosynthesis and Metabolism Treating Human African Trypanomiasis (HAT)

Currently, the only polyamine biosynthetic inhibitor that has been registered for HAT treatment is the irreversible ODC inhibitor DFMO [66]. It is mostly administered in combination with other drugs (nifurtimox, suramin, melarsoprol) depending on the type of Trypanosomes (*T. gambiense* or *T. rhodesiense*) and the disease state of infection (presence or absence of central nervous symptoms). Generally, DFMO is used intravenously for late-stage *T. gambiense* infection. However, the drug regimen is complicated and hampers patients’ compliance due to the high dosing course of 200 mg/kg for 7 days. DFMO also targets ODC from humans. However, selective toxicity to the enzyme from the parasite is achieved due to differences in intracellular turnover rates [67]. Hence, pentamidine is still recommended for the first stage of *T. gambiense* infection. Drug development for polyamine biosynthetic inhibitors in Trypanosomes is hampered by the fact that apart from the core polyamine pathway, a salvage pathway exists. Trypanosomal DHS has been suggested as a target for inhibitor development since it consists of two enzyme paralogs to form the active site [68] which differs significantly from DHS of the human host. Inhibitor development against the dead site NAD^+^ pocket will probably improve selectivity toward the parasitic enzyme but will not circumvent the problems caused by the salvage pathway providing the resource for spermidine. Thus, a combined therapy against two targets is necessary.

## 6. Conclusions and Perspectives

Collectively, this review highlights the importance of the triamine spermidine and its metabolites in the most severe human infections caused by pathogenic viruses and parasitic protozoa. For viral infections such as SARS-CoV-2, Ebola, and HIV-1, it seems a promising pharmacological strategy to stabilize Spd metabolism in the infected host cell. This has been successfully performed in preclinical studies of SARS-CoV-2 infection by spermidine administration to rescue imbalances of EIF-5A formation and autophagy. Disruption of modified EIF-5A expression and EIF-5A-mediated translation of certain viral mRNAs with small molecules might be a way to achieve a broad-spectrum antiviral therapy in the treatment of Ebola and HIV-1 infection. A therapeutic option is more complicated for parasitic diseases such as malaria and Trypanosomiasis since both parasites can utilize spermidine salvage pathways from the human host. Therefore, combined therapies with small next-generation molecules that interfere with spermidine metabolism and transport might be an alternative.

## Data Availability

Not applicable.
